# Nurturing Success: E-Learning readiness and academic self-efficacy in nursing students

**DOI:** 10.1186/s12912-024-02125-2

**Published:** 2024-07-19

**Authors:** Heba Emad El-Gazar, Mohamed Ali Zoromba, Sara Mohamed Fayed, Ahmed Loutfy, Ali A. Elzieny, Amina Elzeiny, Sameer A. Alkubati, Mahmoud Abdel Hameed Shahin, Marwan Altheeb, Ahmed Hashem El-Monshed

**Affiliations:** 1 Department of Nursing Administration, Faculty of Nursing, Port Said University, Port Said, Egypt; 2 Department of Nursing, College of Applied Medical Sciences, Prince Sattam Bin Abdulaziz University, Al-Kharj, Saudi Arabia; 3 Department of Pediatric Nursing, Faculty of Nursing, Port Said University, Port Said, Egypt; 4 Department of Nursing, College of Health Sciences, University of Fujairah, Fujairah, United Arab Emirates; 5 Department of Pediatric Nursing, Faculty of Nursing, Beni-Suef University, Beni-Suef, Egypt; 6 Boston University School of Medicine, Boston Medical Center, Boston, USA; 7 Department of Medical-Surgical Nursing, College of Nursing, University of Hail, Hail, Saudi Arabia; 8 Department of Nursing, Faculty of Medicine and Health Sciences, Hodeida University, Hodeida, Yemen; 9 Nursing Department, Prince Sultan Military College of Health Sciences, Dhahran, Saudi Arabia; 10 Department of Nursing, College of Health and Sport Sciences, University of Bahrain, Manama, Bahrain; 11 Department of Psychiatric and Mental Health Nursing, Faculty of Nursing, Mansoura University, Mansoura, Egypt

**Keywords:** Academic achievement, Academic self-efficacy, Nursing students, Readiness for E-Learning

## Abstract

**Background:**

As nursing education embraces e-learning as a vital component of its pedagogical approach, understanding the interplay between students’ readiness for E-learning and their academic self-efficacy becomes imperative in nurturing successful learning outcomes amidst evolving educational paradigms.

**Purpose:**

This study aimed to explore the relationship between e-learning readiness, academic self-efficacy, and the academic achievement of nursing students within the dynamic educational environment.

**Design:**

This study employed a cross-sectional design.

**Methods:**

A total of 208 nursing students were recruited through convenience sampling at the end of the second semester in 2022. The evaluation included the utilization of Grade Point Average, the Online Learning Readiness Scale, and the Academic Self-Efficacy Scale.

**Findings:**

Previous research has indicated a significant positive correlation between academic achievement and readiness for e-learning, suggesting that higher levels of readiness for e-learning among nursing students lead to improved academic achievement (*p* ≤ 0.001). Additionally, the findings of the current study revealed a notable positive correlation between academic achievement and academic self-efficacy (*p* ≤ 0.001).

**Conclusion:**

This study provides valuable insights into the critical role of academic self-efficacy and e-learning readiness in enhancing academic achievement among nursing students.

## Introduction

Amidst the global spread of COVID-19, the World Health Organization (WHO) declared it a pandemic on March 11, 2020 [1, 2]. This declaration catalyzed significant disruptions across various facets of human existence, particularly in the realm of education [3]. In several countries, the educational system was forced to cease ordinary instructional activities and shut down [4]. Consequently, electronic learning (E-learning) emerged as a vital solution to meet the urgent need for a viable framework ensuring both safety and uninterrupted learning for students. E-learning encompasses a spectrum of digital technologies and media-supported activities [5].

In response to the paradigm shift precipitated by the pandemic, e-learning has undergone a notable transformation, emerging as a ubiquitous practice within educational institutions [6]. E-learning readiness, which pertains to individuals’ proficiency in engaging with electronic and multimedia training tools, plays a pivotal role in improving the overall effectiveness of learning processes. During the initial stages of the transition to e-learning prompted by the pandemic, students faced challenges such as technological limitations and unreliable internet connectivity. E-learning readiness encompasses several dimensions, including access to technology, proficiency in navigating digital platforms, acceptance of e-learning methods, and any required training [7].

A crucial aspect of online education effectiveness is academic self-efficacy, representing students’ confidence in their ability to succeed in their studies. This encompasses various forms, including technology, academic, E-learning system, and computer self-efficacy, all impacting student achievement in digital education [8–12]. Students’ readiness for e-learning during the COVID-19 pandemic is influenced by factors such as initial preparedness, e-learning self-efficacy, and learning autonomy [13, 14]. Despite previous skepticism, the pandemic prompted a shift in attitudes towards online education, leading academic institutions to adopt e-learning to enhance accessibility and convenience for students [15, 16].

E-learning offers numerous advantages, including flexibility, cost-effectiveness, resource accessibility, session recording, constant updates, and 24 h availability [17, 18]. However, challenges such as high implementation costs, limited computer accessibility, copyright awareness among educators, internet issues, and inadequate computer skills remain barriers to widespread adoption [17].

In the transition to remote learning, educators and students increasingly turned to electronic and virtual educational resources, including digital tools and E-learning systems [19]. In Egypt, the Egyptian Knowledge Bank (EKB) emerged as a significant online library of learning materials, provided free of charge to all Egyptians by the government since 2016, particularly crucial during the pandemic [20, 21]. EKB is a cornerstone of E-learning for undergraduates, graduates, and researchers [21], complemented by educational reforms in Egypt, including the introduction of technology such as locally built tablets and laptops [22].

Despite the importance of virtual classrooms, many experts are skeptical that students have the skills necessary to succeed in a digital classroom [23]. Many authors found that the primary cause of failure in implementing E-learning was a lack of preparation [24, 25]. Thus, assessing student readiness through gap analysis is essential, followed by innovative strategies to enhance E-learning integration [26, 27]. A study conducted by Chen et al. [28] to assesses the impressions of E-learning tactics during the Covid-19 pandemic indicated that a student’s academic achievement declined when in-person training was replaced by virtual training. Another Saudi Arabian study conducted during the Covid-19 outbreak demonstrated that the allure of online courses is not universal [29]. In contrast, a study revealed that web-based online instruction was a feasible alternative to traditional classroom instruction [30]. Furthermore, the growth of online medical education was accompanied by the students’ demand for more preparedness [31].

A cross-sectional study revealed the impacts of a higher frequency of not adopting E-learning due to a lack of preparedness plus additional technology features that substantially impact the study population. Construed mental preparedness considers one’s potential to assimilate E-learning [32]. Therefore, the adoption of emerging technology-based methods and an individual’s self-efficacy are the most influential variables influencing the expansion of E-learning. Students’ perceptions of preparedness were reported identically in numerous experiments conducted under diverse conditions [33, 34]. Another study found a significant positive association between self-efficacy and the success of e-learning [35].

The integration of e-learning into nursing education has become increasingly essential, driven by advancements in technology and evolving educational paradigms. As such, understanding nursing students’ readiness and attitudes toward e-learning is paramount for effective educational strategies. Additionally, the COVID-19 pandemic has underscored the importance of home-based e-learning and the need to explore factors influencing students’ engagement in this mode of learning. A recent study in Egypt investigated the relationship between nursing students’ readiness and attitudes toward e-learning, with a focus on the mediating role of self-leadership. The findings revealed that self-leadership significantly influenced students’ attitudes and readiness for e-learning, highlighting the importance of self-directed learning skills in the e-learning environment [36].

Another study aimed to assess e-learning preparedness among nursing students, considering demographic and academic factors. The study identified gender disparities in motivation and online comfort, along with associations between academic factors and e-learning preparedness. The findings underscored the need for further research to understand these demographic influences on e-learning readiness among nursing students [37]. Moreover, Jiang et al. [38] explored the associations between self-control, self-efficacy, and demographic characteristics with home-based e-learning behavior among nursing and midwifery undergraduates during the COVID-19 pandemic. The study identified self-control, self-efficacy, university location, grade level, and perceived health status as predictors of home-based e-learning behavior among nursing undergraduates, emphasizing the importance of individual attributes in shaping e-learning engagement.

Despite the broader acknowledgment of e-learning’s prominence in education [6], a critical gap persists in understanding the nuanced challenges confronted by nursing students during this transition. The swift transition to e-learning, prompted by the pandemic, saw educators and students grapple with various tools and platforms, including video conferencing tools, learning management systems, and massive open online courses [39]. However, while the adoption of e-learning has become standard practice [6], a gap exists in our understanding of how nursing students coped with specific technological challenges, such as poor internet connections and reliance on tools like Zoom and Microsoft Office during the initial phases [40].

In light of the unexplored challenges faced by nursing students in adapting to the dynamic realm of e-learning, this study is dedicated to deepening our understanding of their experiences. Specifically, our aim is to uncover the intricate connections between e-learning readiness, academic self-efficacy, and academic achievement. Recognizing the complexities inherent in the transition to e-learning modalities within nursing education, this study seeks to achieve the following objectives:

Firstly, we endeavor to assess the level of e-learning readiness among nursing students, examining factors such as technological proficiency, self-regulated learning skills, and access to essential resources.

Secondly, we aim to investigate the influence of academic self-efficacy on nursing students’ engagement with e-learning platforms and their perceived ability to excel academically within this context.

Lastly, we aim to explore the relationships between e-learning readiness, academic self-efficacy, and academic achievement among nursing students. Our focus lies in identifying potential predictors and moderators of success in online learning environments.

By delineating these specific research objectives, our intention is to provide a clear and focused overview of the aims and intentions of our study. Through a systematic exploration of e-learning readiness, academic self-efficacy, and their implications for academic achievement, we aspire to contribute valuable insights to the field of nursing education. Furthermore, this research seeks to add empirical evidence to the ongoing discourse surrounding effective strategies for optimizing e-learning experiences and fostering student achievement in nursing education. In presenting a focused exploration of these research objectives and their underlying rationale, we aim to offer a clear roadmap for our investigation and underscore the significance of our study within the broader context of educational research and practice.

## Methods

### Study design and setting

The research was carried out through a cross-sectional survey utilizing quantitative data collection and analysis techniques. Adhering to the reporting guidelines outlined in the STROBE (Strengthening the Reporting of Observational Studies in Epidemiology) Statement for observational studies, the study followed a rigorous methodological approach [41].

### Participants and sampling

The study was conducted on undergraduate nursing students attending a public nursing college in Egypt. This study’s participants were from various online communities (Facebook, WhatsApp, and Telegram). This platform selection was informed by the widespread use of these social media channels among the target population, ensuring efficient outreach and engagement. Undergraduate nursing students at the college had established dedicated social media groups, each corresponding to a specific academic level. These groups served as platforms for both informal and formal communication among students, fostering a sense of community. Leveraging these existing online communities, the research team opted for an online questionnaire as the primary data collection method.

The sample size was determined by performing a power analysis with the regression analysis software G*Power version 3.1.9.7. [42] with 95% confidence, 5% alpha, and 0.15 suggested medium effect size [43], and 11 predictors (academic self-efficacy and readiness for e-learning and nine demographics), given a minimum sample size of 178. Extra 53 students were assessed to be recruited to compensate for the used data collection method and an estimated dropout rate of 30% [44]. Two hundred-eight students responded and completed the survey.

Data collection employed a convenience sampling strategy, chosen to reach a diverse pool of nursing students. Participants included undergraduate nursing students from the college, encompassing various academic levels and study fields. The academic levels represented in the study included first, second, third, and fourth-year students. Study fields were categorized into medical, practical, and theoretical. Furthermore, participants’ device usage, including mobile, tablet, and PC or laptop, was considered. Intern students were deliberately excluded from the study sample as they typically do not utilize E-learning for academic purposes.

The data collection was conducted through the secure online platform Google Forms. The survey link was shared on social media between June 19 and July 12, 2022, spanning three weeks to allow for a comprehensive and varied response. Despite sharing the survey link on social media platforms, confidentiality was upheld through several measures. Firstly, the survey did not collect any personally identifiable information such as names or contact details. Instead, participants were only asked to provide demographic information relevant to the study’s objectives. Secondly, the survey platform utilized, Google Forms, ensured data encryption and secure transmission, safeguarding participants’ responses from unauthorized access. In general, the survey took its participants at most fifteen to twenty minutes to finish. Only those who fulfilled the eligibility requirements could take part in the surveys.

Firstly, prior to data collection, participants were explicitly informed about the study’s focus on academic-related activities within their nursing college community. The survey introduction emphasized the research’s objective of exploring e-learning readiness and academic self-efficacy among nursing students, thereby encouraging participation from individuals actively engaged in academic pursuits. Additionally, the survey questions specifically addressed participants’ experiences, attitudes, and behaviors related to e-learning and academic activities, reinforcing the study’s academic focus. Moreover, the recruitment process leveraged existing social media groups dedicated to academic discussions and resource sharing among nursing students at the college. These groups served as platforms for both formal and informal academic communication, indicating their relevance to participants’ academic endeavors. Furthermore, the survey link was shared within these academic-focused social media groups, ensuring that it reached individuals with an interest in academic-related topics and activities. Overall, these measures were implemented to target participants who regularly engage with social media for academic purposes, thereby enhancing the study’s relevance and validity in investigating e-learning readiness and academic self-efficacy within the nursing college community.

To ensure that all participants enrolled in the target courses were included in the study, several steps were taken to verify their affiliation with the nursing college. Participants were required to provide information about their current academic program, including their enrollment status and course details, during the survey process. Additionally, participants were asked to confirm their enrollment in nursing courses at the public nursing college in Egypt, thereby ensuring that only eligible individuals were included in the study. Furthermore, demographic information collected from participants, such as age, gender, academic level, and other relevant characteristics, provided additional insights into the participant pool. Specifically, the sample comprised undergraduate nursing students from various academic levels. This diverse representation ensured that the sample captured a broad spectrum of the target population and facilitated a comprehensive analysis of e-learning readiness and academic self-efficacy among nursing students.

### Data collection tools

This study employed well-established scales from previous research. Arabic-language online forms were used to collect data. There were three parts to the survey. In the first segment, students’ demographic information was collected. This included their gender, age, academic level, residence, family income, experience with e-learning, device use, single-room status, and most recent semester’s grade point average (GPA). In this study, the term ‘academic achievement’ is operationally defined as the Grade Point Average (GPA) attained by students, which serves as a quantitative measure of their overall academic achievement. The second segment utilized an adapted and validated version of the Online Learning Readiness Scale (OLRS) originally developed by Hung et al. [45]. Comprising 18 items distributed across five dimensions—computer/internet self-efficacy (3 items), self-directed learning (5 items), learner control (3 items), motivation for learning (4 items), and online communication self-efficacy (3 items)—the OLRS employed a 5-point Likert-type scale, ranging from 1 (strongly disagree) to 5 (strongly agree). To create composite scores, the responses for three items within each subscale were averaged, where a higher score indicated greater levels of online learning readiness. The third component involved the Academic Self-Efficacy Scale (ASE), a brief assessment tool with nine items measured on a 7-point Likert-type scale ranging from 1 (Very Untrue) to 7 (Very True). Participants expressed their confidence levels in various academic tasks on this scale, adapted from the work of Chemers et al. [46]. The Arabic version of ASE demonstrated high internal consistency, as indicated by Cronbach’s alpha (α = 0.925) in a study by Almohazie [47].

### Questionnaire’s translation, its validity, and reliability

The process of questionnaire translation and back translation was meticulously executed to ensure the linguistic accuracy and cultural relevance of the instruments used. Initially, the survey was translated from English to Arabic using the forward-and-backward translation technique. This involved two independent translators translating the questionnaire from English to Arabic, followed by another two translators translating it back from Arabic to English. Subsequent adjustments were made to align the translated version with Egyptian norms and cultural nuances. The iterative nature of this translation process allowed for thorough scrutiny of linguistic nuances and cultural sensitivities, thus enhancing the questionnaire’s comprehensibility and appropriateness for the target population. Additionally, to assess the expert validity of the questionnaires, the translated version was made available to nursing professionals, who provided feedback on its applicability, usability, and overall impact. This step aimed to gather insights from individuals with expertise in the field to ensure that the questionnaire effectively captured the intended constructs and was suitable for the context of the study. The feedback obtained from these experts was carefully considered, and necessary revisions were made to refine the questionnaire further. Furthermore, a pilot study involving only 20 nursing students was conducted. The duration of the pilot study spanned from 20th May to 30th May 2022 to thoroughly capture diverse perspectives. By elucidating measurement fit and specifying content in various constructs, the pilot study helped researchers boost reliability and get closer to face and content validity [48]. The final analysis did not use the data from the pilot study. Cronbach’s alpha was computed using SPSS version 26, yielding results between 0.7 and 0.81 for each OLRS subscale and 0.84 for ASE.

### Data analysis

Version 29 of SPSS was employed to conduct comprehensive statistical analyses of the collected data. Descriptive statistics, including percentages and frequency counts, were utilized to summarize all categorical variables, providing a clear overview of the distribution of responses within each category. For continuous variables, such as age and academic achievement, mean and standard deviation (SD) were calculated to characterize the central tendency and variability of the data.

To examine potential differences between groups, a t-test for independent samples was employed for comparing means between two groups, while analysis of variance (ANOVA) was utilized for comparing means across multiple groups. These parametric tests were chosen based on the assumption of normality and homogeneity of variances within groups, which were assessed through visual inspection of histograms and Levene’s test, respectively.

Furthermore, associations between continuous variables were explored using the Pearson correlation coefficient (r). This measure of linear relationship between two variables was selected to assess the strength and direction of associations, with values ranging from − 1 to 1 indicating negative and positive correlations, respectively.

Given the study’s aim to predict academic achievement, multiple regression analysis was deemed more appropriate than other statistical methods. This approach allowed us to examine the simultaneous effects of multiple predictor variables on the outcome variable of academic achievement. Assumptions of linear relationship, normality of residuals, homoscedasticity, and absence of multicollinearity were assessed to ensure the validity of regression analysis results. To address multicollinearity, tolerance values above 0.1 and variance inflation factor (VIF) below 10 were considered acceptable, indicating the absence of high multicollinearity among predictor variables. These diagnostic tests provided assurance regarding the reliability of the regression model. Finally, statistical significance was determined using a conventional threshold of alpha (α) level set at 0.05. Results with *p*-values less than 0.05 were considered statistically significant, indicating a low likelihood of observing the obtained results by chance alone.

## Results

Table 1 shows no difference in studied variables e.g. academic achievement, academic self-efficacy, and readiness for e-learning based on the sociodemographic data of participants.

**Table 1:** Difference of studied variables according to their sociodemographic data (*n* = 208)

Variables		Frequencies		Academic achievement (GPA)		Academic self-efficacy		Readiness for e-learning	
		No	%	Mean (SD)	Sig	Mean (SD)	Sig	Mean (SD)	Sig
Sex	Male	118	56.7	3.2 (0.4)	t = 1.313	34.8 (9.3)	t = 0.493	55.48 (5.4)	t = 0.239
	Female	90	43.3	3.3 (0.3)		35.5 (9.1)		55.66 (5)	
Residence	Urban	125	60.1	3.2 (0.4)	t = 1.056	34.5 (8.8)	t = 1.202	55.5 (4.8)	t = 0.264
	Rural	83	39.9	3.3 (0.4)		36.0 (9.6)		55.7 (5.8)	
Family income	Satisfactory	149	71.6	3.2 (0.4)	t = 0.910	35.0 (9.1)	t = 0.257	55.5 (5.2)	t= 0.26 9
	Unsatisfactory	59	28.4	3.3 (0.4)		35.4 (9.4)		55.7 (5.2)	
Having single room	Yes	119	57.2	3.2 (0.4)	t = 1.550	34.8 (9.1)	t = 0.586	55.2 (5.0)	t = 1.05
	No	89	42.8	3.3 (0.4)		35.5 (9.2)		56.0 (5.4)	
Academic Level	First	36	17.3	3.2 (0.4)	F = 0.181	35.1 (9.1)	F = 0.255	56.3 (5.0)	F = 0.861
	Second	74	35.6	3.2 (0.3)		34.5 (8.9)		54.9 (4.8)	
	Third	41	19.7	3.2 (0.4)		36.0 (9.7)		56.2 (5.8)	
	Fourth	57	27.4	3.2 (0.4)		35.3 (9.2)		55.4 (5.4)	
Study field	Medical	52	25.0	3.3 (0.4)	F = 1.588	36.4 (8.9)	F = 0.766	56.1 (5.0)	F = 0.378
	Practical	56	26.9	3.2 (0.5)		34.3 (9.4)		55.4 (5.1)	
	Theoretical	100	48.1	3.2 (0.4)		34.9 (9.2)		55.3 (5.4)	
Device used	Mobile	37	17.8	3.3 (0.3)	F = 1.246	35.8 (9.5)	F = 0.458	55.9 (5.5)	F = 0.600
	Tablet	84	40.4	3.3 (0.4)		35.5 (9.2)		55.9 (5.2)	
	PC or laptop	87	41.8	3.2 (0.4)		34.4 (9.1)		55.1 (5.0)	
E-learning Experience	Yes	163	78.4	3.2 (0.4)	t = 0.193	35.1 (9.0)	t = 0.029	55.5 (5.2)	t = 0.125
	No	45	21.6	3.2 (0.4)		35.1 (9.8)		55.6 (5.3)	

t = independent sample t test, F = ANOVA test

Table 2 presents descriptive statistics for the variables in our study. The mean age of participants was 19.73 years. GPAs had a mean of 3.22. Regarding psychological and educational aspects, participants had a mean computer/internet self-efficacy of 10.50. Self-directed learning had a mean of 15.70. Learner control had a mean of 7.80. Motivation for learning had a mean of 11.83. Online communication self-efficacy had a mean of 9.73. Academic self-efficacy had a mean of 35.10. Lastly, readiness for e-learning had a mean of 55.56. These statistics offered a concise summary of the participants’ characteristics and formed the basis for our analysis. Participants exhibited moderate levels of computer/internet self-efficacy, self-directed learning, learner control, motivation for learning, online communication self-efficacy, academic self-efficacy, and readiness for e-learning. These findings suggest that students generally possess a reasonable level of confidence in utilizing technology for learning purposes and demonstrate a proactive approach to their studies.

**Table 2:** Descriptive statistics of the studied variables

Studied Variables	Mean	Median	SD	Min	Max
Age	19.73	19.00	1.40	18.00	22.00
GPA	3.22	3.30	0.40	2.40	3.90
Computer/internet self-efficacy	10.50	10.00	2.12	6.00	15.00
Self-directed learning	15.70	15.00	2.29	10.00	20.00
Learner control	7.80	8.00	1.57	5.00	11.00
Motivation for learning	11.83	12.00	1.35	10.00	15.00
Online communication self-efficacy	9.73	10.00	1.21	7.00	12.00
Academic self-efficacy	35.10	35.00	9.17	18.00	52.00
Readiness for e-learning	55.56	55.00	5.19	43.00	68.00

Table 3 reveals significant and positive correlations between readiness for E-learning and academic self-efficacy with academic achievement (*r* = 0.507, *P* ≤ 0.001 and *r* = 0.310, *P* ≤ 0.001). The strength of these correlations underscores the importance of both e-learning readiness and academic self-efficacy in predicting students’ academic success. Additionally, academic achievement exhibits significant and positive correlations with all domains of readiness for E-learning (*P* < 0.005) indicating that various aspects of students’ preparedness for online learning contribute to their overall academic achievement. Similarly, the positive correlations (*P* < 0.005) between academic self-efficacy and readiness for e-learning further emphasize the interconnectedness of these constructs and their collective impact on student outcomes. Importantly, there were no significant correlations found between students’ age and any of the variables under investigation.

**Table 3:** Correlation matrix between studied variables (*N* = 208)

Studied Variables		1	2	3	4	5	6	7	8	9
1) Academic achievement	r	1								
	P									
2) Computer/internet self-efficacy	r	0.189**	1							
	P	0.006								
3) Self-directed learning	r	0.180**	0.283^**^	1						
	P	0.009	0.000							
4) Learner control	r	0.502**	0.130	0.169^*^	1					
	P	0.000	0.062	0.014						
5) Motivation for learning	r	0.444**	0.120	0.185^**^	0.523^**^	1				
	P	0.000	0.085	0.007	0.000					
6) Online communication self-efficacy	r	0.349**	0.082	0.060	0.474^**^	-0.023	1			
	P	0.000	0.238	0.387	0.000	0.741				
7) Readiness for e-learning	r	0.507**	0.623^**^	0.671^**^	0.679^**^	0.545^**^	0.432^**^	1		
	P	0.000	0.000	0.000	0.000	0.000	0.000			
8) Academic self-efficacy	r	0.310**	0.577^**^	0.405^**^	0.306^**^	0.303^**^	0.139^*^	0.619^**^	1	
	P	0.000	0.000	0.000	0.000	0.000	0.045	0.000		
9) Age	r	-0.052	-0.020	-0.017	0.048	-0.012	-0.022	-0.009	-0.050	1
	P	0.459	0.775	0.807	0.493	0.859	0.756	0.892	0.470	

**Correlation is significant at the 0.01 level (2-tailed)

*Correlation is significant at the 0.05 level (2-tailed)

Moving to Table 4, the results of the multiple regression analysis provide deeper insights into the predictors of academic achievement among nursing students. Academic self-efficacy and readiness for e-learning emerged as significant predictors, collectively explaining 36% of the variance in students’ academic achievement. Notably, specific domains within readiness for e-learning, such as motivation, online communication, and learner control, exerted the most substantial influence on academic achievement (F = 18.894, *P* < 0.001). Notably, motivation, online communication, and learner control exerted the most substantial influence on students’ academic achievement (β = 0.322, 0.256, and 0.175, respectively). These findings suggest that students’ beliefs in their academic abilities and their preparedness for e-learning play critical roles in determining their success in academic endeavors.

Overall, the results of our study underscore the importance of fostering e-learning readiness and academic self-efficacy among nursing students. Educators and policymakers should consider implementing interventions aimed at enhancing students’ confidence in utilizing technology for learning purposes, promoting self-directed learning skills, and cultivating a supportive online learning environment. By addressing these factors, educational institutions can better support students’ academic success and ensure positive learning outcomes in the context of e-learning environments.

**Table 4:** Results of a multiple linear regression analysis academic achievement of the studied students (*N* = 208)

Predicators	B	SE(B)	β	t	*P* value
Constant	0.610	0.329	-	1.851	0.066
Computer/internet self-efficacy	0.010	0.013	0.051	0.736	0.463
Self-directed learning	0.005	0.011	0.027	0.440	0.660
Learner control	0.045	0.021	0.175	2.160	0.032
Motivation for learning	0.096	0.022	0.322	4.446	0.000
Online communication self-efficacy	0.085	0.023	0.256	3.714	0.000
Academic self-efficacy	0.004	0.003	0.083	1.094	0.275

*R* = 0.614, R^2^ = 0.377; ΔR^2^ = 0.358; F = 8.083

SE standard error, β standardized regression coefficient

## Discussion

This study delved into the intricate interplay between e-learning readiness, academic self-efficacy, and the academic achievement of nursing students within the ever-evolving educational landscape. The findings underscored the preparedness of nursing students for e-learning, evident in their motivation for online learning, adeptness in computer/Internet self-efficacy and online communication, self-directed learning tendencies, and willingness to engage with peers in virtual settings. These insights shed light on nursing students’ positive perceptions of e-learning, highlighting their satisfaction with this innovative approach and the collaborative dynamics it fosters. Such favorable reception can be attributed to the heightened flexibility and convenience offered by e-learning modalities compared to traditional face-to-face instruction.

Our findings align with prior research emphasizing the advantages of swiftly transitioning to e-learning platforms [49, 50], and the growing preference for such modes among nursing students [51]. Previous studies have underscored the benefits of this shift, with students reporting enhanced levels of readiness for e-learning [49, 50]. Moreover, recent investigations have highlighted a heightened inclination towards e-learning among female nursing students, with significant associations observed across various preparatory subdomains [51]. Moreover, numerous studies indicated that most nursing students in Saudi Arabia [52], Egypt [53], and India were pleased with E-learning during the COVID-19 pandemic [54]. In contrast to Bdair’s [55] findings, our study suggests that nursing students demonstrated significant motivation for virtual classes. This disparity could stem from differences in study populations, regional disparities in virtual learning platform implementation, or evolving student attitudes towards online education. Moreover, contextual factors such as institutional support, resource quality, and exposure duration to virtual learning environments may have influenced student motivation. Further exploration of these nuanced factors could offer deeper insights into the determinants of nursing students’ motivation in the realm of virtual education.

The variations observed in our study’s findings compared to previous research may be attributed to several factors. Firstly, differences in the study population, including demographic characteristics such as age, gender, and academic background, could influence students’ perceptions and experiences with e-learning. Additionally, regional differences in the implementation of virtual learning platforms and the availability of technological resources may contribute to disparities in students’ readiness and preferences for e-learning. Moreover, contextual factors such as institutional support, the quality of online resources, and the duration of exposure to virtual learning environments may play pivotal roles in shaping students’ motivation and satisfaction with e-learning. Furthermore, the evolving nature of students’ attitudes towards online education over time, particularly in response to external factors such as the COVID-19 pandemic, could impact their perceptions and experiences with e-learning. Therefore, further research exploring these nuanced aspects and conducting comparative analyses across different populations and contexts is essential to gain a more comprehensive understanding of the factors influencing nursing students’ motivation and satisfaction in the context of virtual education.

The present study underscores a significant positive association between academic achievement and e-learning readiness, indicating that higher levels of preparation among nursing students correlate with improved academic success. This link highlights the crucial role of learners’ acceptance of modern technology-based methods and their academic self-efficacy in shaping academic outcomes. This may be explained by the data collected between June 19 and July 12, 2022, two years after the outbreak began. Students enhance their E-learning preparedness and attain elevated levels of academic self-efficacy and academic achievement as they gain experience and training in utilizing educational technology over time.

Consistent with previous research, our study aligns with prior findings emphasizing the importance of e-learning preparedness for academic success [56, 57]. These studies showed that semester-prior GPA or academic achievement is a strong predictor of preparation for E-learning during situations like COVID-19. In addition, a prior study found the factors influencing the success of remote education; using multiple linear regression, GPA was a predictor for distance education readiness score [58]. Multiple studies have demonstrated substantial correlations between students’ academic achievement and their readiness to use e-learning [59–61].

Moreover, research from Arabic contexts has underscored the positive impact of e-learning implementation strategies on students’ academic achievement. Similarly, various studies have consistently highlighted the efficacy of the e-learning model in facilitating access to extensive information while offering enhanced learning flexibility that caters to individual differences, ultimately contributing to improvements in academic achievement [62–66].

The findings of our study highlight a significant positive association between academic achievement and academic self-efficacy, indicating that students’ belief in their academic abilities correlates with improved achievement. Enhanced academic self-efficacy fosters increased confidence among students, which in turn influences various aspects of their academic journey. This link can be attributed to factors such as heightened confidence, resilience in facing challenges, intrinsic motivation, effective cognitive and metacognitive skills, and positive impacts on overall well-being. Recognizing and nurturing academic self-efficacy is paramount for educators and institutions aiming to bolster students’ academic success and well-being.

Furthermore, this improved confidence translates into the ability to set goals, overcome challenges, and approach academic endeavors with a positive mindset, potentially leading to improved academic achievement. Previous research has highlighted the diverse impact of academic self-efficacy on individual development, emphasizing its connection to individuals’ perceptions of their abilities, including critical thinking, goal setting, and resilience, which can positively influence academic success [67, 68].

Additionally, supporting our findings, another study indicates a favorable correlation between self-efficacy and grade point average, suggesting that academic achievement tends to be higher among students exhibiting greater intrinsic motivation for knowledge and a higher-class rank [69]. Similarly, an Egyptian study discovered a statistically significant positive correlation between self-efficacy and academic achievement among nursing students [70]. However, it is important to note that the current study’s results may not align completely with a prior study that did not demonstrate a significant correlation between nursing students’ academic achievement and self-efficacy [71].

In general, this study corroborates the findings of numerous studies that investigated the role of self-efficacy in determining the academic achievement of undergraduate university students [72–75]. These studies revealed a variation in the effect of general self-efficacy on the grade point average. According to multiple regression analyses of the current study, academic self-efficacy and e-learning preparedness were significant predictors of academic success. In contrast, another study’s regression analysis revealed that academic self-efficacy did not predict academic achievement [76].

The outcomes of this study underscore a significant and positive correlation between academic self-efficacy and readiness for E-learning. This linkage suggests that self-efficacy plays a pivotal role in preparedness for E-learning. The OLRS, utilized in this study, incorporates five dimensions, with two of them specifically addressing computer/internet self-efficacy and online communication self-efficacy. Consistent with this, a prior study found that as students’ academic self-efficacy levels increased, so did their readiness for learning and motivation to learn [77].

In contrast to findings aligning with the present study, another research effort revealed that nursing students exhibited low readiness ratings for learning and did not experience an improvement in their self-efficacy [78]. This discrepancy was also echoed in another study with similar findings [79]. It is important to acknowledge the diversity in results across studies, which may be influenced by various contextual factors and individual differences among student populations.

The current study provided valuable insights into the factors influencing the academic achievement of nursing students in the context of E-learning readiness and academic self-efficacy. The significant predictors, academic self-efficacy, and readiness for E-learning collectively elucidate a considerable portion of the variance in students’ academic achievement. Understanding the specific dimensions contributing significantly is crucial for tailoring interventions and support mechanisms.

Amid the challenging circumstances of the COVID-19 pandemic, the substantial influence of motivation on academic achievement becomes increasingly evident. High motivation levels are crucial for sustaining students’ commitment to academic pursuits amidst disruptions and uncertainties in traditional learning environments. This finding aligns with existing literature underscoring the importance of motivation in navigating unconventional learning contexts [49, 50]. Additionally, online communication emerges as a significant predictor, emphasizing its critical role in fostering collaborative learning environments, particularly in the absence of traditional face-to-face interactions. The study highlights the importance of honing online communication skills to create a sense of community among students engaged in remote learning, reflecting the heightened reliance on virtual communication tools observed globally during the pandemic.

Furthermore, the substantial influence of learner control underscores the importance of students actively managing and directing their online learning experiences. This dimension reflects adaptability and resilience in navigating diverse digital platforms, teaching styles, and assessment methods. Students who demonstrate a higher degree of control over their learning journey tend to exhibit enhanced academic achievement, aligning with the broader understanding that empowering students with control fosters a sense of ownership and engagement in their educational endeavors.

The observed differences and similarities between our study’s findings and previous research can be attributed to various factors, including methodological differences, sample characteristics, and contextual factors. Firstly, variations in the measurement and operationalization of academic self-efficacy and readiness for e-learning across studies may contribute to disparities in the observed outcomes. Different instruments or scales used to assess these constructs could yield divergent results, affecting the comparability of findings. Additionally, differences in sample characteristics, such as the demographic composition of the student population and their prior experiences with e-learning, could influence the strength and direction of associations between academic achievement, self-efficacy, and e-learning readiness. Moreover, contextual factors, including institutional support for e-learning implementation, the quality of online resources, and the duration of exposure to virtual learning environments, may impact students’ perceptions and experiences with e-learning, consequently influencing their academic achievement. Furthermore, variations in the timing of data collection and the duration of exposure to e-learning platforms may also contribute to differences in the observed outcomes, particularly in response to external factors such as the COVID-19 pandemic. Despite these variations, consistent findings across studies underscore the importance of academic self-efficacy and readiness for e-learning in shaping students’ academic achievement, highlighting the need for tailored interventions and support mechanisms to enhance students’ preparedness and confidence in navigating virtual learning environments.

In summary, the results underscore that beyond the technical aspects of E-learning, motivational factors, proficiency in online communication, and the ability to exert control over one’s learning journey are critical determinants of academic success. These insights can inform targeted interventions and support strategies aimed at enhancing these specific dimensions, ultimately improving nursing students’ academic outcomes during periods of educational disruption.

### Strengths and limitations

This study significantly enriches literature by delving into the intricate interplay between academic self-efficacy, e-learning readiness, and academic achievement among nursing students, particularly in the context of the COVID-19 pandemic. A limitation lies in the exclusive use of quantitative methods, neglecting individual perspectives and narratives. Future research should incorporate qualitative instruments, such as semi-structured interviews, to enhance the depth of understanding. The study’s outcomes may be influenced by participants’ diverse backgrounds, and the majority being first- and second-year students may limit generalizability. Lastly, sampling bias could have occurred due to the convenience sampling method employed, which may limit the generalizability of the results to broader populations of nursing students. Additionally, reliance on self-report questionnaires for data collection introduces the possibility of response bias, as participants may provide socially desirable responses or inaccurately report their experiences and perceptions. Furthermore, the cross-sectional design of the study limits our ability to establish causal relationships between variables and precludes the examination of temporal trends over time.

## Conclusion

In conclusion, our study underscores the critical importance of academic self-efficacy and e-learning readiness in shaping the academic achievement of nursing students during the pandemic. The results emphasize the need for tailored interventions and support mechanisms to enhance students’ preparedness for online learning, ultimately fostering academic success. This study holds significance for educators and policymakers, offering a unique perspective on effectively preparing nursing students for E-learning, ensuring high academic achievement. Insights from this study can guide interventions to enhance students’ online readiness, thereby improving academic outcomes during the ongoing pandemic and future challenges. To enhance academic achievement, instructors should assess students’ academic self-efficacy for E-learning, providing training in digital tools to overcome potential obstacles. Improved preparation for online learning can be achieved through computer and Internet instruction, increased access to technology resources, and assessing students’ technological capabilities beforehand.

Institutions play a crucial role in enhancing students’ e-learning readiness and academic self-efficacy to improve academic outcomes. Firstly, institutions can provide comprehensive orientation programs and training sessions to familiarize students with e-learning platforms and technologies, thereby boosting their confidence and competence in utilizing these tools effectively. Additionally, offering ongoing support services, such as academic advising and counseling, can help students develop resilience and problem-solving skills, enhancing their academic self-efficacy. Moreover, fostering a supportive learning environment that encourages collaboration, feedback, and self-reflection can promote students’ sense of belonging and motivation, contributing to their overall academic success.

In future research, it would be valuable to investigate the long-term effects of interventions designed to enhance students’ e-learning readiness. By conducting longitudinal studies, researchers can assess the sustainability of these interventions and their impact on academic outcomes over extended periods. Additionally, exploring the role of institutional support in e-learning success warrants further investigation. Research could delve into how different forms of institutional support, such as academic advising and technical assistance, influence students’ e-learning experiences, academic self-efficacy, and ultimately, their academic achievement. These avenues of inquiry would provide valuable insights into enhancing students’ preparedness for e-learning and optimizing their academic success in virtual learning environments.

## Data Availability

The datasets generated during and analyzed during the current study are not publicly available due to confidentiality agreements, but are available upon reasonable request from the corresponding author.
